# Case Report: Refractory Ventricular Fibrillation Resolved by Double External Defibrillation and Beta Blockade

**DOI:** 10.5811/cpcem.3861

**Published:** 2025-04-01

**Authors:** Humza Khan, Jennifer Campoli, Susan Wojcik

**Affiliations:** Upstate Medical University, Department of Emergency Medicine, Syracuse, New York

**Keywords:** refractory ventricular fibrillation, double external defibrillation, beta blockade, esmolol, defibrillation

## Abstract

**Introduction:**

The mortality rate for refractory ventricular fibrillation (RVF) can be up to 97%. There is no widely accepted treatment plan for this stage of ventricular fibrillation besides the standard combination of defibrillation, amiodarone, and epinephrine. One novel approach that has been documented in a select few cases since 2015 is the combination of double external defibrillation (DED) and esmolol-induced beta blockade.

**Case Report:**

We report the case of a 65-year-old man who presented with RVF after collapsing at work. Upon the simultaneous administration of two defibrillators with a combined shock of 400 joules and 35 milligrams of the beta blocker esmolol, the patient regained pulse and began blinking. He was discharged from the hospital after seven days and walked out of the clinic.

**Conclusion:**

This case continues the trend of several case reports since 2015 that have featured beta blockade and double external defibrillation as a viable solution to refractory ventricular fibrillation. Since there is limited quantifiable data on the efficacy of this treatment, future studies should aim to evaluate whether the combination of DED and beta blockade has the potential to become the new standard in treating RVF over a broader patient population.

## INTRODUCTION

Ventricular fibrillation (VF) is the leading cause of sudden cardiac death in patients with myocardial infarction, accounting for about 70% of mortalities.^1^ Ventricular fibrillation is characterized by a period of sporadic electrical output interfering with the process of ventricular excitation, which physically manifests in the heart rate becoming too high to competently pump blood. The patient often dies in minutes.^2^ With early intervention, typically consisting of cardiopulmonary resuscitation (CPR) and external defibrillation, many patients can have a prognosis comparable to those with myocardial infarction who have not experienced VF.^3^ Patients may also experience refractory ventricular fibrillation (RVF), where a return of spontaneous circulation (ROSC) cannot be established within 10 minutes despite three attempts at defibrillation and the administration of 300 milligrams (mg) of amiodarone and 3 mg of epinephrine. In these cases, the mortality rate can be up to 97%.^4^ At this stage of cardiac arrest, the use of double external defibrillation (DED) and beta blockade may be considered, although the research to quantitatively support both treatments in RVF is limited.^5^,^6^ The following is a case of RVF that responded to the use of DED in combination with beta-blocker therapy.

## CASE REPORT

A 65-year-old man presented to the emergency department (ED) for a cardiac arrest witnessed outside the hospital. The patient was at work with his colleagues when he collapsed and was noted to be pulseless. Bystander CPR was initiated until emergency medical services arrived. During transportation to the hospital via ambulance, an initial VF rhythm was found; subsequently, the patient was defibrillated a total of five times and given six doses of epinephrine 1 mg. In addition, he received bicarbonate, calcium, and 450 mg of amiodarone prior to arrival. During transport to the ED, he had a brief, five-minute period of ROSC but lost his pulse again prior to ED arrival. During ROSC, his blood pressure was 132/112 millimeters of mercury, his respiration rate was 20 breaths per minute on pulse oximetry with a saturation of 89%, and his heart rate was 69 beats per minute. Total time between the onset of cardiac arrest and arrival at the ED was 40 minutes.

Upon arrival, the patient had a Lund University Cardiopulmonary Assist System (LUCAS) in place and was placed onto a stretcher. For two minutes CPR was performed, during which he was given 1 mg of epinephrine. He was intubated during the first pulse check, and the rhythm check showed VF on the monitor. Cardiopulmonary resuscitation was restarted, and two Zoll defibrillators were attached to the patient and charged to 200 joules (J) each. The two defibrillations were delivered simultaneously, with a combined output of 400 J. Then CPR was resumed, and 35 mg of esmolol (approximately 0.5 mg/kilogram) was administered. Shortly after, the patient began blinking, and during the subsequent pulse check he was noted to have a strong carotid pulse.

The patient was placed on continued mechanical ventilation and brought to the cardiac catheterization lab. He was found to have significant occlusions in both the proximal left anterior descending artery (LAD) and the proximal left circumflex artery (LCA), requiring four stents to be placed in the LAD and two in the LCA. He then was put under targeted temperature management and started on vasopressors from which he was weaned over two days. On day four he was extubated. On day seven he was discharged and able to walk out of the hospital. During his one-week follow-up visit, the patient stated that he felt great and was back to his normal, active lifestyle and did not experience any of his original symptoms. He also did not need or want cardiac rehabilitation services, and even quit smoking.

## DISCUSSION

Current guidelines for managing VF emphasize the significance of early intervention with the use of CPR and an automated external defibrillator as being crucial to increasing chances of survival. In fact, in cases of out-of-hospital cardiac arrest where the victim receives early bystander CPR, defibrillation, or both, the incidence of brain damage and death from any cause has been found to be significantly lower.^7^ Given the rapid and early care given to the patient in our case, it is likely that this intervention prevented him from garnering significant neurological injury and was certainly a contributing factor to his full recovery.

The management of RVF is less clear. One of the approaches to treating RVF is to use novel defibrillation strategies. A 2022 study found that in RVF patients, both double sequential external defibrillation (DSED) and vector-change (VC) defibrillation were associated with significantly higher rates of survival to hospital discharge than standard defibrillation. Moreover, DSED but not VC defibrillation was associated with a higher percentage of patients with a favorable neurological outcome.^8^

**CPC-EM Capsule**

What do we already know about this clinical entity?

*Ventricular fibrillation is a common cause of cardiac death; current advanced cardiovascular life support guidelines recommend cardiopulmonary resuscitation, defibrillation, epinephrine, and antiarrhythmics*

What makes this presentation of disease reportable?

*This patient’s refractory ventricular fibrillation (RVF) was resolved by a combination of double external defibrillation and beta blockade*.

What is the major learning point?

*The novel combination of double defibrillation and esmolol can be effective in treating RVF, leading to recovery*.

How might this improve emergency medicine practice?

*These findings could help redefine RVF management, improving survival and outcomes by integrating novel defibrillation techniques and pharmacotherapy*.

There are several theories that may explain the relative success of DSED in RVF patients: the power theory, the setting up theory, and the multiple vector theory. According to the power theory, the use of more energy allows for more complete recruitment and conversion of the patient’s myocytes out of RVF rhythm. If using this approach, the two shocks must be delivered simultaneously, as performed in this particular case. According to the setting up theory, the two shocks must be performed with a deliberate pause between. The first current lowers the defibrillation threshold, allowing for a higher chance at success converting all the remaining myocytes with the second shock. Finally, the multiple vector theory works in tandem with both the two aforementioned theories, as it simply entails the application of multiple defibrillation pads on a patient, thereby increasing the number of vectors that can be used to conduct current to the myocardium.^5^

## CONCLUSION

The administration of the beta blocker esmolol is useful in high-acuity cases such as those of RVF patients, as the drug exhibits a very rapid onset with a nine-minute half-life.^9^ In a 2016 study by Lee et al, sustained ROSC was far more common in RVF patients treated with esmolol than in controls; additionally, short- and long-term survival and neurological outcomes were more than twice as good in the esmolol group.^10^ Despite the documented benefits of both esmolol and double external defibrillation as separate treatments for RVF patients, there is limited literature available on the integration of both of these strategies in clinical cases. Since this combination of treatment was first proposed in a 2015 paper by McGovern and McNamee, there have been several case reports documenting the success of using double sequential external defibrillation and esmolol-induced beta blockade in RVF patients.^9^,^11^,^12^ Future studies should aim to bring quantifiable data to the table regarding this combined intervention, with the end goal of creating a more proven, standardized approach to recognizing and treating RVF in patients with myocardial infarction.
